# Interaction of CSFV E2 Protein with Swine Host Factors as Detected by Yeast Two-Hybrid System

**DOI:** 10.1371/journal.pone.0085324

**Published:** 2014-01-08

**Authors:** Douglas P. Gladue, Ryan Baker-Bransetter, Lauren G. Holinka, Ignacio J. Fernandez-Sainz, Vivian O’Donnell, Paige Fletcher, Zhiqiang Lu, Manuel V. Borca

**Affiliations:** 1 Plum Island Animal Disease Center, Agriculture Research Service, United States Department of Agriculture, Greenport, New York, United States of America; 2 Department of Pathobiology and Veterinary Science, University of Connecticut, Storrs, Connecticut, United States of America; 3 Plum Island Animal Disease Center, Department of Homeland Security, Greenport, New York, United States of America; Kantonal Hospital St. Gallen, Switzerland

## Abstract

E2 is one of the envelope glycoproteins of pestiviruses, including classical swine fever virus (CSFV) and bovine viral diarrhea virus (BVDV). E2 is involved in several critical functions, including virus entry into target cells, induction of a protective immune response and virulence in swine. However, there is no information regarding any host binding partners for the E2 proteins. Here, we utilized the yeast two-hybrid system and identified fifty-seven host proteins as positive binding partners which bound E2 from both CSFV and BVDV with the exception of two proteins that were found to be positive for binding only to CSFV E2. Alanine scanning of CSFV E2 demonstrated that the binding sites for these cellular proteins on E2 are likely non-linear binding sites. The possible roles of the identified host proteins are discussed as the results presented here will be important for future studies to elucidate mechanisms of host protein-virus interactions during pestivirus infection. However, due to the limitations of the yeast two hybrid system, the proteins identified is not exhaustive and each interaction identified needs to be confirmed by independent experimental approaches in the context of virus-infected cells before any definitive conclusion can be drawn on relevance for the virus life cycle.

## Introduction

Classical swine fever virus (CSFV) and bovine viral diarrhea virus (BVDV) are highly contagious diseases of swine and bovine, respectively. Both are small, enveloped viruses with a positive-sense, single-strand RNA genome and are classified as members of the pestivirus genus within the *Flaviviridae* family [1]. The approximately 12.5-kb pestivirus genome contains a single open reading frame that encodes a polyprotein composed of 3,898 amino acids that ultimately yields 11 to 12 final cleavage products (NH2-N^pro^-C-E^rns^-E1-E2-p7-NS2-NS3-NS4ANS4B-NS5A-NS5B-COOH) through co- and post-translational processing of the polyprotein by cellular and viral proteases [2]. Structural components of the virions include the capsid (C) protein and glycoproteins: E^rns^, E1 and E2. E1 and E2 are anchored to the envelope at their carboxyl termini, and E^rns^ loosely associates with the viral envelope [3]–[5]. E1 and E2 are type I transmembrane proteins with an N-terminal ectodomain and a C-terminal hydrophobic anchor [5]. E2 is considered essential for CSFV replication, as virus mutants containing partial or complete deletions of the E2 gene are nonviable [6]. E2 is the most immunogenic of the CSFV and BVDV glycoproteins [3], [7], [8], inducing neutralizing antibodies and protection against lethal CSFV or BVDV challenge. E2 has been implicated, along with E^rns^ [9] and E1 [10], in viral adsorption to host cells; indeed, chimeric pestiviruses exhibit infectivity and cell tropism phenotypes consistent with those of the E2 gene donor [7], [11]. Modifications introduced into this glycoprotein appear to have an important effect on CSFV virulence [12]–[16]. It is evident that pestivirus E2 plays many critical roles. Recently the E2 protein of BVDV has been crystallized, revealing a three domain structure. Domains I and II are similar to Ig-like domains and domain III is a series of three small β-sheet modules; this structure is believed to be similar to CSFV E2 by prediction analysis [17], [18]. Although it is obvious that E2 plays a critical role during virus infection, there is no direct evidence of any host binding partners to either CSFV or BVDV E2. To advance the current understanding of the functions of the pestivirus E2 protein, we attempted to identify host proteins that directly interact with the E2 protein of CSFV or BVDV by means of the yeast two-hybrid system using custom swine and bovine cDNA libraries. Results indicate that both CSFV and BVDV E2 interact with fifty-seven different host proteins, while two additional proteins interact solely with CSFV E2. Attempts to map any of the host protein binding sites within the CSFV E2 protein by using a poly-alanine scanning mutagenesis approach suggests that the host proteins bind a structurally non-linear portion of E2. The possible roles of the identified host proteins are discussed. Identification of host proteins directly interacting with pestivirus E2 may significantly improve the understanding of the role of E2 during infection and virulence. However, each interaction identified here needs to be confirmed by an independent experimental approach in the context of virus-infected cells before any definitive conclusion can be drawn on relevance for the virus life cycle.

## Materials and Methods

### Development of the cDNA Libraries

A porcine primary macrophage cDNA expression library was constructed (Clontech, Mountain View, CA) using monocytes/macrophages obtained from healthy CSFV-free swine exactly as previously described [19]. Macrophage cultures were prepared from defibrinated swine blood. Total RNA was extracted from adherent cells using an RNeasy Mini kit (Qiagen, Valencia, CA). Contaminant genomic DNA was removed by DNase treatment using TURBO DNA-*free* (Ambion, Austin, TX). After DNase treatment, genomic DNA contamination of RNA stocks was assessed by real-time PCR amplification targeting the porcine β-actin gene. RNA quality was assessed using RNA Nano Chips on an Agilent Bioanalyzer 2100 (Agilent Technologies, Santa Clara, CA). Cellular proteins were expressed as GAL4-AD fusion proteins while CSFV E2 was expressed as a GAL4-BD fusion protein. Following a similar procedure, a bovine cDNA expression library was constructed (Clontech, Mountain View, CA) using different tissues from healthy non-infected bovine [20].

### Library Screening

The GAL4-based yeast two-hybrid system was used for this study [21], [22]. The ‘bait’ protein, CSFV Brescia E2 protein (amino acid residues 1–342) or BVDV NADL E2 protein (amino acid residues 1–342), was expressed with an N-terminus fusion to the GAL4 Binding Domain (BD). As ‘prey’, the previously described swine macrophage cDNA library and bovine cDNA library containing proteins fused to the GAL4 Activation Domain (AD) was used. Screening was done as previously described [19]. Sequencing of the identified library clones was performed by the dideoxynucleotide chain-termination method [23]. Sequencing reactions were prepared with the Dye Terminator Cycle Sequencing Kit (Applied Biosystems, Foster City, CA). Reaction products were sequenced using an ABI PRISM 3730xl automated DNA sequencer (Applied Biosystems, Foster City, CA). The identified sequence was checked to be in-frame with the GAL4-AD and the NCBI BLAST algorithm was used to identify the host protein.

### PANTHER Classification of Proteins

Positive interacting host proteins that bound CSFV and BVDV E2 proteins were entered into the PANTHER classification program. The resulting classification was used to group proteins by biological process. The PANTHER program [24] can be found at http://www.pantherdb.org/.

### Construction of an Alanine Scanning Mutagenesis Library for CSFV E2

Full-length CSFV E2 from Brescia strain was used as a template in which native amino acids were substituted with alanine, introduced by site-directed mutagenesis using the Quick Change XL Site-Directed Mutagenesis kit (Stratagene, Cedar Creek, TX), performed per manufacturer's instructions. Briefly, the full-length plasmid was amplified by PCR, digested with Dpn1 to leave only the newly amplified plasmid, transformed into XL10-Gold ultra competent cells, and grown on Terrific Broth plates containing ampicillin. Positive colonies were grown for plasmid purification using the Qiagen Maxiprep kit. The full-length E2 was sequenced to verify that only the desired mutation was present in the plasmid. Primers were designed using the Stratagene Primer Mutagenesis program. This program limits a maximum number of amino acid changes, based on primer length and the number of nucleotides that had to be changed for every mutant, setting the basis for deciding on the regions to be mutated. Primers were designed using the manufacturer’s primer design program: https://www.genomics.agilent.com/CollectionSubpage.aspx?PageType= Tool&SubPageType = TolQCPD&PageID = 15.

## Results and Discussion

### Conservation in E2 Amino Acid Sequence among Pestiviruses

**Figure 1:**
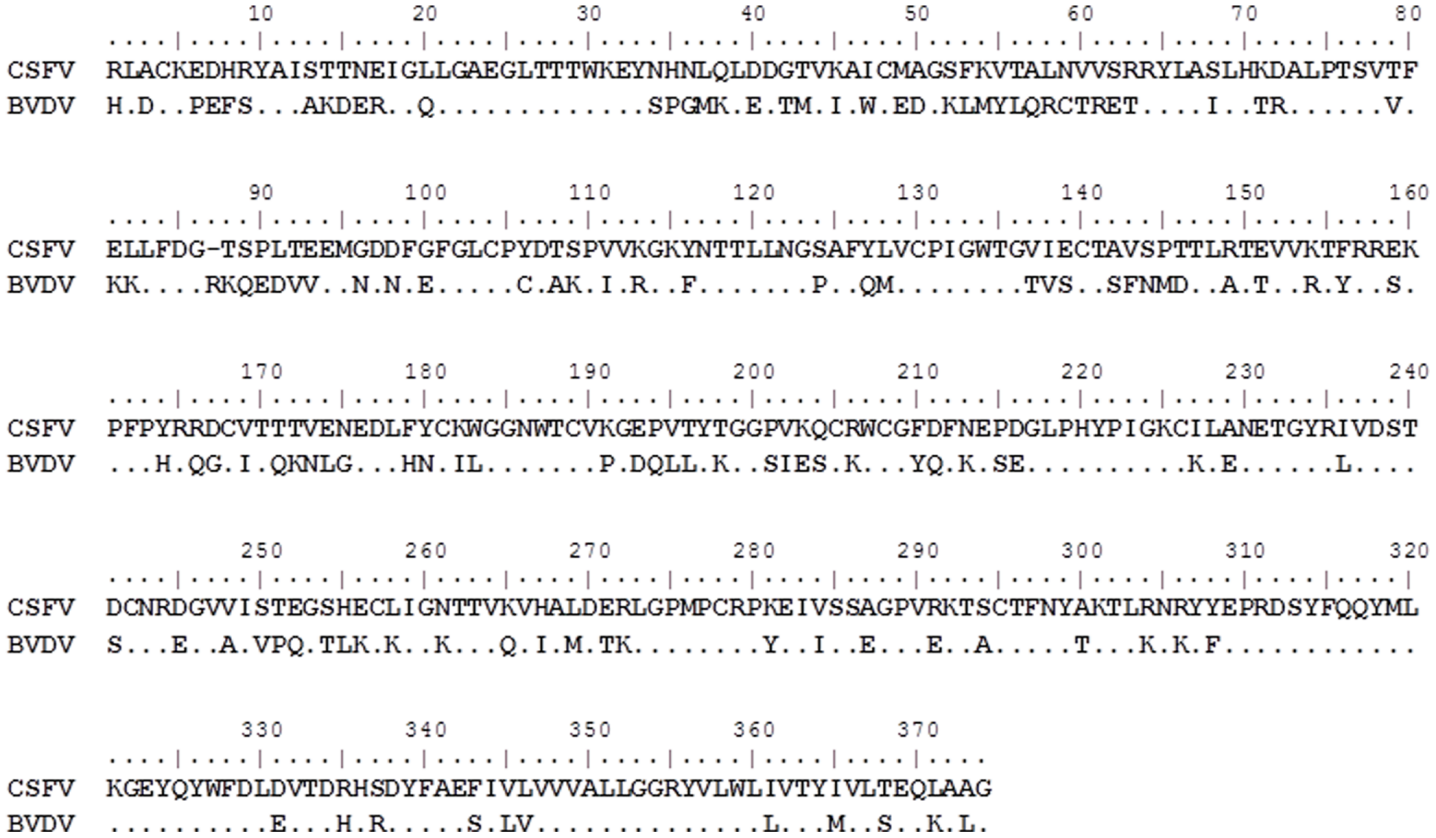
Multiple sequence alignment using Bioedit software was performed using CSFV E2 from the Brescia isolate (GenBank Accession #AF091661) and the BVDV E2 from the NADL isolate (GenBank Accession #AJ133738.1).

A sequence alignment between CSFV E2 from the Brescia isolate (GenBank Accession #AF091661) or the BVDV E2 from the NADL isolate (GenBank Accession #AJ133738.1) was performed, and a comparison between the amino acid sequence of these two proteins revealed a 65% identity and a 74% similarity depending on which isolate was being compared [17] (Fig. 1). Exchanging the E2 protein from one pestivirus with another pestivirus E2 results in changes to cell tropism *in vitro* [11], [25], suggesting that although there are differences in sequence between the different E2 proteins, they share enough common features that they can be exchanged and allow for virus replication. However, it has been reported that a chimeric BVDV virus with the E2 protein of CSFV was unable to cause disease in swine although it was shown to efficiently grow in swine cells, suggesting that the E2 proteins of CSFV and BVDV have enough similarity to be exchanged for growth in cell culture, but additional factors are needed to cause disease in the non-native host [25].

### Specificity of E2 Proteins in the Yeast Two-hybrid

The E2 protein from either CSFV or BVDV was cloned into the yeast two-hybrid vector with the N-terminus of E2 fused to the binding domain (BD) of gal4. Both the CSFV and BVDV E2 proteins tested lacked the C-terminal transmembrane domain to allow the protein to enter the nucleus, a requirement for the yeast two-hybrid model. The level of background activity for the E2 proteins was assessed by co-expressing E2-BD with the T-antigen coupled to the AD (TAg-AD), a common negative control protein used in the yeast two-hybrid. Both pestivirus E2s were determined to have a low degree of background when grown on media lacking adenine and histidine, two nutritional markers that are driven by the gal4 promoter only in the presence of binding between the two proteins being tested. P53-BD and TAg-AD were included as a positive control for the yeast two-hybrid (data not shown). Cells were grown on media lacking only plasmid selection nutritional markers (tryptophan and leucine), allowing growth in the absence of protein binding, as a control. Our results show that there was no background growth on media lacking adenine and histidine, and that both CSFV E2 and BVDV E2 are suitable for screening a host protein library.

### Yeast Two-hybrid Screening Results

To identify host cellular proteins that interact with the CSFV or BVDV E2 proteins, both proteins were screened against both a custom bovine and a custom swine cDNA library. Approximately 1×10^7^ independent yeast colonies were tested, representing approximately 3-fold library saturation, as both libraries were determined to contain approximately 3×10^6^ independent clones. Positive colonies were selected for growth on selection media (-Leu/-Trp/-His/-Ade). Plasmids were recovered in *E.coli* and sequenced. In-frame proteins were retested for specificity to CSFV E2, BVDV E2, BD only, and Lam-BD, a negative control commonly used in the yeast two-hybrid. Fifty-seven proteins were identified as positive binding partners to both the CSFV E2 and the BVDV E2 protein (Table 1).

**Table 1:** Proteins that interact with CSFV+BVDV E2.

Gene symbol	Gene Name	Gene symbol	Gene Name
ACADM*	acyl-CoA dehydrogenase, C-4 to C-12 straight chain	NDUFS1	NADH dehydrogenase (ubiquinone) Fe-S protein 1,75 kDa
ALDH7A1	alpha-aminoadipic semialdehyde dehydrogenase-like	NUP43*	nucleoporin 43 kDa
CAPZA2	capping protein (actin filament) muscle Z-line, alpha 2	PELI1*	E3 ubiquitin-protein ligase pellino homolog 1
CCDC115*	coiled-coil domain-containing protein 115-like	PHC3	polyhomeotic homolog 3 (Drosophila)
CCDC80	coiled-coil domain containing 80	POFUT2*	protein O-fucosyltransferase 2
CCT7	chaperonin containing TCP1, subunit 7	POMP *	proteasome maturation protein
CEP57	centrosomal protein of 57 kDa	PPT1	palmitoyl-protein thioesterase 1
CFP	complement factor properdin	PRDX3*	thioredoxin-dependent peroxide reductase, mitochondrial
CTSB*	cathepsin B	PRMT10*	protein arginine methyltransferase 10
CTSH*	cathepsin H	QARS	glutaminyl-tRNA synthetase
DCTN6	dynactin 6	RMD5*	required for meiotic nuclear division 5 homolog B
DOCK7*	dedicator of cytokinesis 7	SDCBP*	syndecan binding protein (syntenin)
EIF6	eukaryotic translation initiation factor 6	SERTAD1*	SERTA domain-containing 1
EMID1*	EMI domain containing 1	SERTAD3*	SERTA domain-containing protein 3
FANCF*	fanconi anemia, complementation group F	SIVA1*	apoptosis-inducing factor
FANCL*	fanconi anemia, complementation group L	SPG11	spastic paraplegia 11
FBLN5	fibulin 5	TARDBP*	TAR DNA-binding protein 43
FLNA	filamin A, alpha	TBL1XR1	transducin (beta)-like 1 X-linked receptor 1 isoform 2
GCA*	grancalcin-like	TCEB1	transcription elongation factor B (SIII), polypeptide 1
GCH1	GTP cyclohydrolase 1	TOR1AIP2	torsin A interacting protein 2
HGPRT*	hypoxanthine phosphoribosyltransferase 1	TRAPPC2	trafficking protein particle complex 2
HMCN1	hemicentin 1	TRAPPC8	trafficking protein particle complex 8
KLHL20	kelch-like 20 isoform CRA_c	TXN2	thioredoxin
LGALS3	lectin, galactoside-binding, soluble, 3	UPF0712*	C7orf64 homolog
LTBP1	latent transforming growth factor beta binding protein 1	UPF1	regulator of nonsense transcripts homolog
MAGOHB	mago-nashi homolog B	UXT	Ubiquitously-expressed transcript
MRPL45	mitochondrial ribosomal protein L45	VCAN*	Versican
NCF2	neutrophil cytosol factor 2		

proteins discovered in the swine library. All other proteins discovered in the bovine library.

### Differential Binding of Host Proteins to Pestivirus E2 Proteins

Despite the number of proteins identified that bound both BVDV E2 and CSFV E2, there were some differential binding partners for these viral proteins. Two of the proteins identified were positive binding partners for CSFV E2 and did not bind BVDV E2 (Table 2). One protein is a member of the heterogeneous nuclear ribonucleoprotein (HNRP) family (HNRPF). HNRPs have been shown to be binding partners for a wide range of viruses, and disruption of this binding or knockdown of HNRPs causes a decrease in viral replication or viral release. For example, HNRNPD has been shown to bind Epstein-Barr Virus-encoded RNA 1 (EBER1) [26] and HNRNPK binds Epstein-Barr virus nuclear antigen 2 (EBNA2) which enhances viral protein LMP2A expression by an unknown mechanism [27]. In Japanese encephalitis virus (JEV) infections, HNRNP A2 has been shown to be important for viral replication, binding both the core protein and the viral RNA [28]. HNRNP H1 also binds the core protein of Hepatitis C virus [29]. During an infection with herpes simplex virus-1, depletion of HNRNP K inhibits viral egress [30]. Although the exact mechanisms are not known for the requirement for HNRP’s that bind viral proteins or viral RNA, it can be hypothesized that viral binding of HNRNPs can regulate both host and viral RNA, and may provide some way for viruses to enhance their own RNA replication and translation, or possibly is a way to prevent host RNA translation during viral infection.

**Table 2:** Host proteins exclusively binding CSFV E2.

Gene symbol	Gene Name
HNRPF*	heterogeneous nuclear ribonucleoprotein F isoform 5
PTBP2*	polypyrimidine tract-binding protein 2

proteins discovered in the swine library.

The other protein that was positive for CSFV E2, polypyrimidine tract-binding protein 2 (PTBP2), is highly homologous to polypyrimidine tract binding protein (PTB) but thought to be located primarily in brain and neural cells. PTBP2 has ribonucleoprotein domains, has been shown to enhance HNRNPH function, and could be involved with other HNRNPs, suggesting both proteins that were positive for CSFV E2, PTBP2 and HNRNPF, could be involved in a similar pathway. Interestingly, these are the only two proteins that were determined to be a positive binding partner for CSFV E2 and did not bind BVDV E2.

### Classification of E2-binding Host Proteins by Biological Process

**Figure 2:**
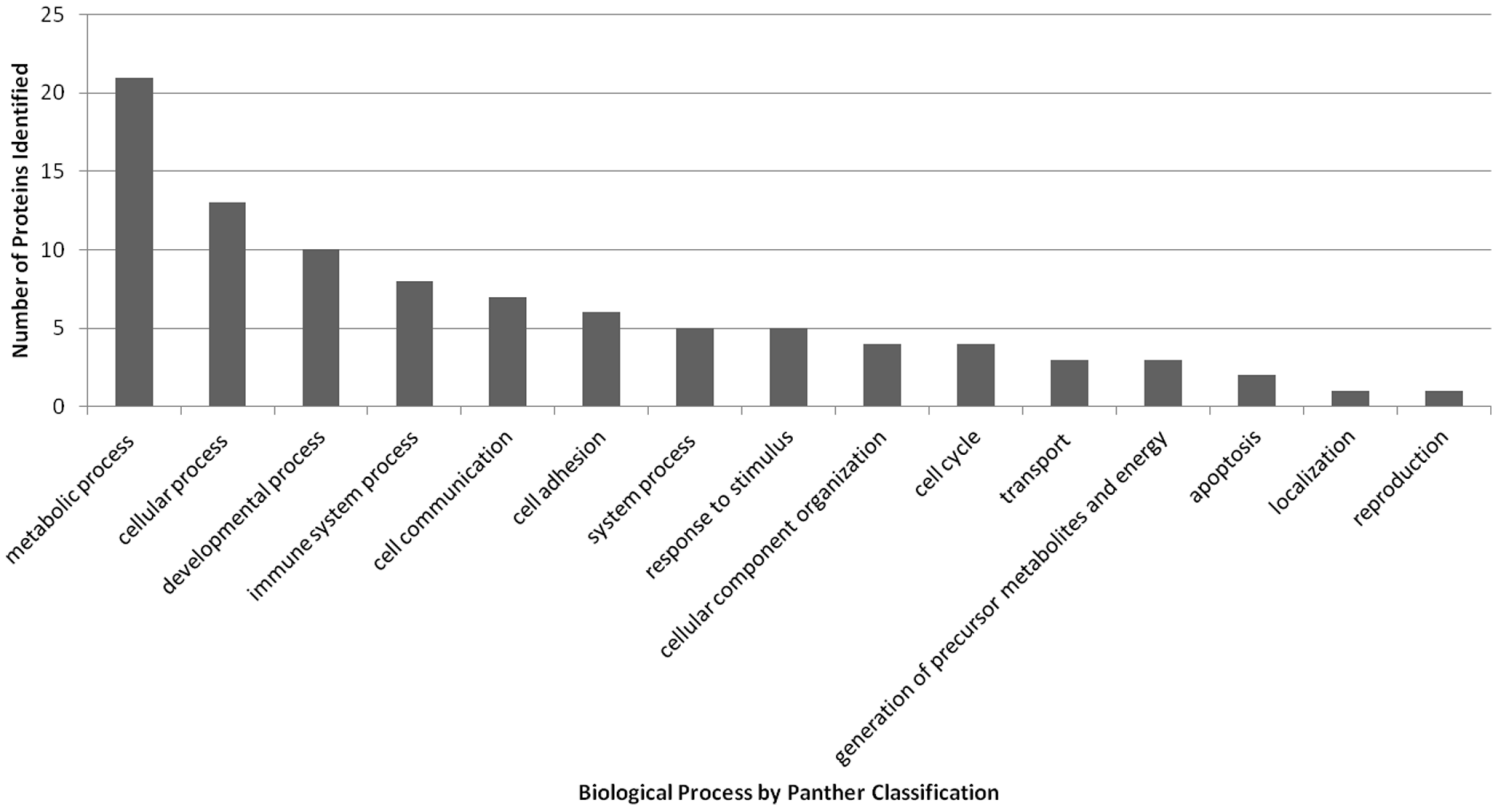
A graphic view of the distribution of E2 positive interacting proteins, as separated into Biological processes, as defined by the PANTHER classification system.

To gain some insight into the potential biological processes that are regulated by pestivirus E2 proteins, we classified all the identified host proteins using the PANTHER (Protein ANalysis THrough Evolutionary Relationships) classification system [24] (Fig. 2). For the proteins that bind both E2 proteins, the PANTHER analysis identified 15 different biological processes defined in Table 3, as the function of a particular protein in the context of a larger network of proteins that interact to accomplish a process at the level of the cell or organism. A brief analysis of these proteins in the context of their biological processes and their hypothetical significance in CSFV replication and virulence are discussed.

**Table 3:** Biological process classification of E2 interacting proteins by PANTHER analysis.

Biological Process	Definition	Proteins Identified
Metabolic processes:	Any process involving chemical reactions and pathways, bywhich living organisms transform chemical substances.	CTSH, GCH1, PPT1, CCDC80, EIF6, PHC3, TCEB1, PRDX3,CCT7, HGPRT, KLHL20, MAGOHB, FBLN5, TXN2, ACADM,DCTN6, QARS, ALDH7A1, PRMT10, TARDBP, UXT
Cellular processes	Any process that is carried out at the cellular level	CCDC80, PHC3, CAPZA2, HMCN1, KLHL20, LGALS3, DOCK7,FLNA, FBLN5, LTBP1, TXN2, VCAN, TARDBP
Developmental Processes:	Any process whose specific outcome is the progression ofan integrated living unit that develops froman initial condition to a later condition	PHC3, CAPZA2, HMCN1, KLHL20, FLNA, MAGOHB, FBLN5,LTBP1, VCAN, TARDBP
Immune System processes:	Any process involved in the development orfunctioning of the immune system	CFP, CTSH, CCDC80, NCF2, PRDX3, LGALS3, FBLN5,TXN2
Cell Communication:	Any process that mediates interactions betweena cell and its surroundings	CCDC80, DOCK7, FLNA, FBLN5, LTBP1,TXN2, VCAN
Cell Adhesion:	Any process involved in the attachment of a cell,either to another cell or to an underlying substrate	CDC80, HMCN1, LGALS3, FLNA, FBLN5,VCAN
System process:	A multicellular organismal process carried out byany of the organs or tissues in an organ system	HMCN1, KLHL20, FLNA, FBLN5, LTBP1
Response to stimulus:	Any process that involves a change in state oractivity of a cell or an organism as a result of a stimulus.	CFP, CTSH, CCDC80, NCF2, TXN2
Cellular componentorganization:	A process that is carried out at the cellular levelwhich results in the assembly, arrangement of constituentparts, or disassembly of acellular component	PHC3, CAPZA2, KLHL20, FLNA
Cell cycle:	The progression of biochemical and morphologicalphases and events that occur in a cell during successivecell replication or nuclearreplication events.	PHC3, FLNA, TXN2, TARDBP
Transport:	Processes involved in the movement of substancesinto, out of, within or between cells, or within amulticellular organism	TRAPPC8, DOCK7, SDCBP
Generation of precursormetabolites andenergy:	The chemical reactions and pathways resulting inthe formation of precursor metabolites, substances fromwhich energy is derived, and any process involved inthe liberation of energy from these substances	NDUFS1, TXN2, ACADM
Apoptosis:	Any process that is involved in a form ofprogrammed cell death	LGALS3, TXN2
Localization:	Any process by which a cell, a substance, ora cellular entity, is transported to, and/or maintained ina specific location	MAGOHB
Reproduction	Processes involved in production by an organismof new individuals that contain some portion of theirgenetic material inherited fromthat organism	MAGOHB

Twenty-one proteins were classified as being involved in PANTHER Metabolic Processes within the cell (Table 3). We, along with others, have previously used microarray analysis to identify subsets of genes in cellular metabolic pathways involved in DNA replication that were down-regulated during CSFV infection [31]–[33]. The host proteins identified in the yeast two-hybrid screening as being part of these metabolic processes are (Table 3). Several of these host proteins have been shown to be involved in the pathogenesis of other viruses. For example, Chaperonin containing TCP1, subunit 7 (CCT7) is a member of the TCP1 Ring complex (TRiC). This complex is involved in correctly folding various proteins, and has been shown to be involved in the replicative process of influenza A virus [34], type D retrovirus [35] and Hepatitis C virus (HCV) [36]. Another protein, DCTN6 (Dynactin 6), is part of the Dynactin complex, which binds cargo (organelles, virus, vesicle, etc) to dynein or kinesin for intracellular transport. This transport system has been implicated as critical for several viruses for cellular entry or cellular escape, including pseudorabies virus (PrV) [37], adenovirus [38] and the human immunodeficiency virus type 1 (HIV-1) [39]. During an Epstein-Barr virus (EBV) infection, the protein UXT (ubiquitously-expressed, prefoldin-like chaperone) is phosphorylated, causing the downregulation of NF-κB transactivation, and may possibly play a critical role in the lytic cycle of EBV. This class of proteins is important to the process of replication and pathogenesis for other viruses, suggesting they could play also an important role in pestivirus virulence.

Thirteen of the host proteins identified by the yeast two-hybrid screening are involved in the PANTHER biological pathway of cellular processes (Table 3). In addition, the PANTHER biological pathway of developmental processes, is represented by 10 of the host proteins identified (Table 3). There is a large amount of overlap in the developmental process and cellular process groups, likely because proteins that are involved in cellular processes can ultimately play a role in organism development; because of this overlap we will discuss the two process groups together. Some of these proteins have been described as playing an important role in viral processes. For example, LGALS3 (lectin galactoside-binding soluble protein 3), is known to be involved in the process of virus entry during viral infection with parvovirus [40] and HSV-1 [41]. In addition, LGALS3 is up-regulated during Junín virus (JUNV) infection although its role during infection is still unknown [42]. Other galectin proteins have been implicated as being important for pathogenesis during viral infection for both HIV [43] and influenza virus [44]. This suggests the possibility that pestiviruses could utilize LGALS3 as part of the mechanism for viral entry; however, further studies would be required to determine the specific role for E2 binding to LGALS3.

Viral subversion of the immune system during the replication of CSFV has been repeatedly shown [45]–[49]; thus, it is no surprise that several of the proteins that were identified in our screen are classified as being part of PANTHER immune system processes (Table 3). CFP, or complement factor properdin, is a regulator of the alternative complement activation pathway and it is a protein target for immune regulation by other viruses. In dengue virus infection, CFP is up-regulated [50], while in HSV-1 the CFP binding site in C3 is blocked by HSV-1 glycoprotein gC [51] and in HIV-1, glycoprotein gp12 has binding affinity to CFP. Many viruses have evolved ways to prevent activation of the complement pathway, and perhaps CSFV and BVDV block this pathway though the interaction between E2 and CFP.

Further analysis of identified host proteins using PANTHER classification schemes revealed host proteins that belong to ten other biological processes, cell communication, cell adhesion, system processes, response to stimulus, cellular component organization, cell cycle; transport, generation of precursor metabolites and energy, apoptosis, localization, and reproduction (Table 3). Some of these proteins overlap in several biological functions as cellular proteins often play multiple roles in multiple pathways. Further studies of the potential effect of these proteins binding to pestivirus E2 proteins will have to be done to gain a better understanding of which cellular pathways E2 may be involved in.

In addition, several proteins were unable to be classified by the PANTHER classification system as belonging to any predefined biological process. Those are host proteins CDC115, CEP57, CTSB, EMID1, FANCF, FANCL, GCA, MRPL45, NUP43, PELI1, POFUT2, POMP, RMD5, SERTAD1, SERTAD3, SIVA1, SPG11, TBL1XR1, TOR1AIP2, TRAPPC2, UPF0712 and UPF1 (Table 1). However, some of these proteins are known to be important during other viral infections. For example, host protein Cathepsin B (CTSB) promotes Heptatis B infection [52], while inhibition of CTSB decreases HIV particle release from the infected cell [53], [54]. Additionally, host protein Fanconi anemia complementation group L (FANCL), was determined to interact with CSFV protein NS2 [55], suggesting the possibility that FANCL could play multiple roles during CSFV infection by binding to different CSFV proteins. Interestingly, now we are reporting an additional member of this family, FANCF (group F), as an additional binding partner for E2. Host protein Nup43, or nuclear pore protein 43 kDA, is a component of the nuclear pore. In polio virus [56], vaccinia virus [57] and HIV-1 [58] infections, the viral proteins have been shown to interact with other nuclear pore proteins to either allow the viral genome to enter the nucleus or to prevent host mRNA from exiting the nucleus, thus preventing host transcription. Additionally, host protein PELI1, or Pellino-1, has been implicated in regulating the epithelial cellular response to rhinoviruses [59], while SIVA1, or apoptosis-inducing factor, has been shown to be required for influenza A virus replication [60] and is important for apoptosis regulation during viral infection by HIV-1 [61], Human papillomavirus-16 [62] and coxsackievirus B3 [63]. A regulator of nonsense transcripts homolog, UPF1, has been implicated in human T-lymphotropic virus type 1 to interact with tax protein, inhibiting nonsense-mediated mRNA decay [64]. During a Rous sarcoma virus (RSV) infection, UPF1 degrades viral RNA in the absence of the RSV stability element RSE, suggesting a role for RSE in avoiding UPF1 degradation. During HIV-1 infection, UPF1 is a component of the HIV-1 ribonucleoprotein (RNP), and expression of UPF1 directly influences HIV-1 RNA expression [65].

It is clear that many of the proteins identified by the yeast two-hybrid system as host proteins that interact with the E2 proteins of CSFV and BVDV are involved in different aspects of viral pathogenesis in other viral infections, strongly suggesting that these proteins could play a role in the pathogenesis of pestiviruses.

### Construction of Poly-alanine Mutant Library

**Figure 3:**
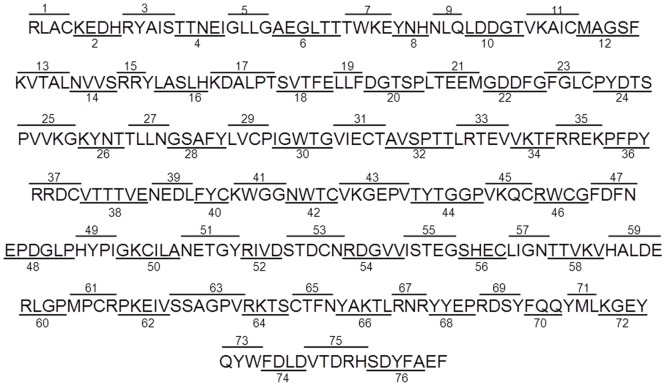
Scheme showing CSFV E2 alanine mutants used in the present study. All indicated residues were mutated to an alanine.

In an attempt to map the binding site(s) for the host proteins that interact with CSFV E2, an alanine scanning mutagenesis approach was used. This particular approach has been used in our laboratory to facilitate elucidation of specific areas within FMDV viral proteins recognized by host proteins [20], [66]. We used site-directed mutagenesis to construct a set of 76 CSFV E2 mutant proteins containing sequential stretches of amino acids where the native amino acid residues were substituted by alanine residues (Fig. 3). The complete set of mutated E2 proteins the yeast two-hybrid system, were assessed for their ability to bind all of the host proteins identified that interacted with native CSFV E2. After testing all of the potential mutants with all of the positive cellular proteins, we were unable to map any of the binding sites, as all of the host proteins were capable of binding all of the E2 alanine mutants. These results suggest that disruption of a single linear amino acid stretch is not sufficient to alter the binding of any of the positive cellular proteins. This was initially an unexpected result; however, the recent determination of the crystal structure of BVDV E2 has demonstrated that the structure of BVDV E2 is a rather complex spatial structure. Therefore, it is possible that most of the host proteins would bind multiple non-linearized arranged residues that would not be disrupted by changing a single linear residue stretch to alanine. To map the residues responsible for host protein binding would require a more complex mutagenesis approach to determine the areas of protein interaction within E2.

## Conclusion

We identified fifty-five host proteins that bind structural glycoprotein E2 of both CSFV and BVDV (Table 1). Two additional host proteins recognized CSFV E2 but did not interact with BVDV E2 (Table 2). Many of the host proteins identified have also been identified by other means to interact with other viruses, and in many cases determined to be important in the process of viral replication and/or pathogenesis. We cannot rule out the limitations of the yeast two-hybrid methodology in regards to the identification of host proteins that may not actually interact with E2 in the infected cells, as our studies determined proteins that were capable of interacting in the yeast two-hybrid system, and not in a virus infected cell. It is also possible, that some host proteins were not identified either due to the host protein not being present in either library or due to the inability of the protein to interact due to the limitations in the yeast two-hybrid system. For example, unidentified protein interactions that may only occur exist within the environment of the infected cells or in the presence of other viral proteins such as proteins that interact with the E2–E1 heterodimer. CD46 the cellular receptor for BVDV E2 [67] was also not detected, most likely due to CD46 being a membrane protein, making it unlikely that it would enter the nucleus in the yeast two-hybrid system. Nevertheless, the identification of cellular factors potentially interacting with structural glycoprotein E2 during virus infection is critical to explaining phenomena known to involve E2 as well as to discover novel roles for E2.

Further work is needed in order to truly evaluate the importance of the interactions described here in regards to their possible roles in virus replication or virus virulence, as our study was limited to identification of proteins in the yeast two-hybrid, and there is the possibility that some of these protein interactions due not occur in cells that are infected with the virus. However, the massive identification of the host proteins potentially interacting with E2, a viral protein that has been shown to be one of the most important proteins for virus virulence is very valuable information and can help us to understand the potential mechanisms of CSFV replication and pathogenesis. In order to determine the significance of each of the host proteins identified here, additional studies are required to explore the role(s) of each of these proteins during pestivirus infection.
